# Droplet Microarray Based on Superhydrophobic-Superhydrophilic Patterns for Single Cell Analysis

**DOI:** 10.3390/microarrays5040028

**Published:** 2016-12-09

**Authors:** Gabriella E. Jogia, Tina Tronser, Anna A. Popova, Pavel A. Levkin

**Affiliations:** Karlsruhe Institute of Technology, Institute of Toxicology and Genetics, Hermann-von-Helmholtz-Platz 1, 76344 Eggenstein-Leopoldshafen, Germany; gabriella@student.sgu.ac.id (G.E.J.); tina.tronser@kit.edu (T.T.); anna.popova@kit.edu (A.A.P.)

**Keywords:** Droplet Microarray, superhydrophobic-superhydrophilic patterning, single-cell analysis, high-throughput screening, screening

## Abstract

Single-cell analysis provides fundamental information on individual cell response to different environmental cues and is a growing interest in cancer and stem cell research. However, current existing methods are still facing challenges in performing such analysis in a high-throughput manner whilst being cost-effective. Here we established the Droplet Microarray (DMA) as a miniaturized screening platform for high-throughput single-cell analysis. Using the method of limited dilution and varying cell density and seeding time, we optimized the distribution of single cells on the DMA. We established culturing conditions for single cells in individual droplets on DMA obtaining the survival of nearly 100% of single cells and doubling time of single cells comparable with that of cells cultured in bulk cell population using conventional methods. Our results demonstrate that the DMA is a suitable platform for single-cell analysis, which carries a number of advantages compared with existing technologies allowing for treatment, staining and spot-to-spot analysis of single cells over time using conventional analysis methods such as microscopy.

## 1. Introduction

For many years, studies of single cells have been under the highlight of research in interdisciplinary biological fields. Analysing single cells versus bulk cell population provides information about the small and concise environment of a single cell, enabling the understanding of complex biological phenomena. As the applications of sciences and technology are becoming more advanced, the single-cell analysis (SCA) is becoming important as a tool for diagnostics, for example, as a prediction model for complex genotype–phenotype interactions and drug-response. In addition, SCA is applied in fundamental studies of the heterogeneity of cell behaviour in cell populations, such as the variance of proliferation rates of cells and the maintenance of the pluripotency in stem cells [1,2,3].

Despite multiple studies have demonstrated heterogeneity within cell populations [4,5], most clinical analyses and diagnostics are performed on bulk cell populations. In this case, there is a risk that potential biomarkers produced by unique individual cell types are being masked by expression profiles of larger subpopulations of cells. In contrast, SCA can provide fundamental information about single cells within heterogeneous cell populations and enable identification of unique biomarkers expressed by single cells [6,7,8,9].

The role of SCA in understanding the mechanisms of carcinogenesis, tumour progression and chemoresistance has been recognized in recent studies [10,11]. The high degree of cellular heterogeneity in tumours often leads to ineffective treatments, due to the fact that some cells respond to the therapy and others develop resistance. SCA can give information on drug response of single cells, therefore, providing a more accurate model for drug response in patients [12,13]. In the field of stem cells research, the SCA can facilitate the identification of factors and pathways that influence pluripotency. In addition, studies based on SCA enable the identification and investigation of factors and pathways that control stem cells differentiation, which is important for fundamental research and for the development of regenerative therapies [14,15,16,17]. In the future, the use of SCA will enrich our fundamental knowledge in biological research, as well as in diagnostics and therapeutics.

Performing SCA is challenging, especially in the field of stem cell research, due to low survival rates of single cells and the absence of appropriate platforms for single-cell culture [18,19]. One of the main limitations of SCA is the minute amount of cell material, such as DNA, RNA or protein contents, which is available for analysis, considering that part of the cell content is being lost during the isolation procedures. This is critical, for example, in single-cell sequencing [20], as well as in other omics analyses.

Most known and recent technologies used for SCA studies include microfluidics, laser microdissection, flow cytometry, manual cell picking and limiting dilution using hand pipettes or robots [21]. While these methods have provided significant information in single-cell studies in the past years, they still face numerous challenges. For example, microfluidic platforms, the most used for single-cell sequencing and metabolic assays, are mainly closed systems, costly and depend on flow control systems [22,23,24]. In addition, microfluidic platforms, including those based on droplet and dielectrophoresis microfluidics, do not provide proper physiological cell environments [25]. This is critical especially for adherent cells, which need a surface to attach to and appear to grow better in conventional culture plates than in droplet emulsions in microchannel [26]. Finally, mentioned platforms usually have to be combined with other platforms, such as microwell plates, to support the analysis system [27,28]. Therefore, effective strategies for SCA studies are being explored. Scientists from different fields and different expertise are working on establishing more suitable methods for culturing and analysing single cells. The ideal platform should be easy to use in a conventional way, enable access to enough material for analysis, be compatible with high-throughput techniques, provide compartmentalisation and be cost-effective.

In this study, we optimized the Droplet Microarray (DMA) platform for SCA. The DMA is a miniaturized biocompatible platform that provides defined confinement of droplets on the surface of glass slides. This is achieved by utilizing a protocol developed by Feng and colleagues [29], which is based on thiol-yne click chemistry for the fabrication of superhydrophilic-superhydrophobic (SH-SL) micropatterns on a thin polymer layer attached to a standard microscope glass slide. This platform provides several advantages including defined nanoliter-sized compartments for culturing cells, easy and fast accessibility of spots and low consumption of samples and reagents, as well as applicability for high-throughput screening applications [30,31,32,33,34,35]. The combination of simple chemistry for the fabrication of patterns and the compatibility with standard laboratory equipment, such as cell culture incubators and screening microscopes, makes it possible to perform high-throughput analysis in a conventional cost-effective way.

On the DMA platform, cells are cultured in individual droplets of nanoliters’ volume confined on superhydrophilic (SL) spots. These droplets are separated from each other by superhydrophobic (SH) borders. This allows culturing single cells in small volumes and in multiple completely isolated confined reservoirs. This miniaturization of the culture reservoirs enables fast and direct follow up of multiple isolated single cells due to small space with minimalized medium use compared to conventional multiwell plates. Small volumes might also be advantageous for culturing single cells because the growth factors and signalling molecules released by the cell are not diluted in large volumes and more likely to promote the growth and proliferation of the same cell. A confined isolated environment excludes any communication and cross-reaction between the spots enabling analysis of single cells without influence of any environmental factors coming from other cells, such as cell–cell communication, signalling and exchange of soluble factors [36]. Therefore, the DMA platform enables observation of single cells under completely isolated conditions, while providing cells with the necessary nutrients and growth factors. In addition, due to the effect of discontinuous dewetting, the DMA platform allows for easy pipetting and pump-free seeding of cells on the whole DMA chip, which contains hundreds of spots per standard microscope glass slide. These unique features of the platform make it compatible with high-throughput without a need for any additional equipment. We envision that the DMA platform can become a pioneering technology for various SCA applications.

This paper focuses on establishing protocols for creating single-cell arrays on the DMA platform and the culture of single cells in individual droplets. We investigated the behaviour of single cells confined in droplets on the DMA and its dependence on different variables of the experimental setup, such as size of the spots, initial seeding density, duration of seeding and medium composition. Proliferation and survival rates of single cells were analysed and compared to those exhibited by multiple cells. We believe that the DMA platform is well suited for SCA and provide proof that this platform offers advantages compared to the platforms currently used for culturing and analysing single cells.

## 2. Materials and Methods

### 2.1. Preparation of the Droplet Microarray (DMA) Slides

The preparation of DMA was adapted from a previously published protocol [29]. Briefly, glass slides (Schott Nexterion, Jena, Germany) were activated by immersing in 1 M sodium hydroxide solution (Merck KGaA, Darmstadt, Germany) for 1 h, followed by immersing it in 1 M hydrochloric acid solution for 30 min. The activated slides were modified by applying 20% v/v ethanol and a 3-(trimethoxysilyl)propyl methacrylate solution (Sigma-Aldrich, Munich, Germany) on the surface of the glass slides, followed by incubation for 30 min. The modification process was repeated 2 or 3 times.

The fabrication of porous polymer layer was done by applying polymerization mixture containing 24 wt.% 2-hydroxyethyl methacrylate (HEMA; Sigma-Aldrich), 16 wt.% ethylene dimethacrylate (EDMA; Sigma-Aldrich), 12 wt.% 1-decanol (Sigma-Aldrich, Munich, Germany), 48 wt.% cyclohexanol (Sigma-Aldrich, Munich, Germany) and 0.4 wt.% 2,2-dimethoxy-2-phenylacetophenone (DMPAP; Sigma-Aldrich) on the modified slides, followed by covering the slide with a fluorinated glass slide. The fluorinated glass slides were prepared by incubating them overnight in a sealed desiccator containing an open vial with trichloro(1*H*, 1*H*, 2*H*, 2*H*-perfluorooctyl)silane (Sigma-Aldrich) at 50 mbar.

The polymerization was done via UV irradiation of the slides, using 260 nm wavelength for 15 min with 10 mW/cm^2^ intensity using a deep-UV collimated light source Model 30 (Optical Associates Inc., San Jose, CA, USA) fitted with a 500 W Hg-xenon lamp (Ushio, Tokyo, Japan). The polymer surface was then rapidly taped to increase the polymer surface roughness. The “taping” was performed by applying adhesive tape (Tesa, Offenburg, Germany) on the polymer surface followed by rapidly removing the adhesive film from the surface.

Polymerized slides were then modified through immersion in a mixture containing 45 mL of dichloromethane (Merck KGaA), 56 mg of 4-(dimethylamino)pyridine (DMAP; Novabiochem, Merck KGaA, Darmstadt, Germany), 111.6 mg of 4-pentanoic acid (Sigma-Aldrich) and 180 µL of *N*,*N*′-diisopropylcarbodiimide (DIC) solution (Alfa Aesar GmbH & Co. KG, Karlsruhe, Germany) under stirring for at least 4 h, followed by washing with ethanol and drying using a nitrogen gun.

The superhydrophobic background was created by applying a 5% v/v perfluorodecanethiol solution (Sigma-Aldrich) in acetone onto the polymer surface, followed by UV-irradiation through a photomask (Rose Fotomasken, Bergisch Gladbach, Germany) with 260 nm wavelength for 1 min with 10 mW/cm^2^ intensity in dark environment, washing with acetone and drying. The superhydrophilic areas (static water contact angle of 5.2°) [29] were created by applying 10% v/v ß-mercaptoethanol (Alfa Aesar) dissolved in 1:1 v/v water and ethanol solution onto the patterned surface, covering it with a quartz microscope slide (Science Services GmbH, Munich, Germany) and irradiating the slides under the same conditions as above. The slides were washed with ethanol, dried using a nitrogen gun and stored until further usage.

### 2.2. Cell Culture on the Droplet Microarray

Stably transfected HeLa cells expressing Green Fluorescent Protein (HeLa-GFP cells; BioCat GmbH, Heidelberg, Germany) were cultured using Dulbecco’s Modified Eagle’s Medium (DMEM) (Gibco, Life Technologies GmbH, Darmstadt, Germany), containing 10% fetal calf serum (FCS; Sigma Aldrich), 1% Penicillin/Streptomycin (Gibco, Life Technologies GmbH) and 1% Blasticidin (Gibco, Life Technologies GmbH) in a CO_2_ incubator (BINDER GmbH, Tuttlingen, Germany) at 37 °C with 5% CO_2_ level. At confluence, cells were washed using phosphate buffered saline (PBS) solution (Gibco, Life Technologies GmbH, Darmstadt, Germany), followed by trypsinization of cells using 0.25% trypsin/EDTA (Gibco, Life Technologies GmbH). Cell subculturing was done every 2–3 days with a ratio of 1 to 7.

Prior to cell seeding, DMA was sterilized with ethanol, dried under a sterile hood (LAB-KITS, Utherm International (H.K) Limited, Hong Kong) for 15 min, incubated in DMEM medium containing 1% FCS, 1% Penicillin/Streptomycin and 1% of Blasticidin for 25 min, followed by air-drying under the hood for 60 min. The superhydrophilic spots on the DMA slide were then coated with 2.2% w/v gelatin in Milli-Q water (Sigma-Aldrich) [34]. The DMA was then kept in the incubator for 60 min and then air-dried under the sterile hood for 45 min or 20 min, for *standard* or *undried* seeding method, respectively.

Cells were seeded onto the DMA slide as described by Popova et al. [35] The DMA slide was placed in a 50 mm petri dish, and 1.4 mL of cell suspension with a defined cell concentration was pipetted onto DMA slide for 45, 60 or 75 s, followed by tilting the slides to form droplets on the superhydrophilic (SL) areas. To avoid evaporation of the droplets, a humidified environment was created by placing the Petri dish containing DMA inside a 100 mm Petri dish containing tissue paper and PBS solution.

### 2.3. Analysis of Cell Distribution

Each field containing 14 × 14 spots (DMA with 1 mm spots), 27 × 27 spots (DMA with 500 µm spots) and 39 × 39 spots (DMA with 350 µm spots) was imaged immediately after seeding using KEYENCE Fluorescence Microscope BZ-9000 (KEYENCE, Osaka, Japan) at 2× magnification using “merge” function of the microscope software BZ II-Viewer (KEYENCE, Osaka, Japan). The initial cell number in the droplets was estimated by manual counting using ImageJ (Version 1.51f, Bethesda, MD, USA) software. Spots were grouped depending on the initial quantity of cells in the droplet. The experiment was repeated 3 times independently, with 9 arrays analysed.

### 2.4. Estimation of Cell Viability and Proliferation Rate

To estimate the viability and proliferation rate of cells, DMA slides with 500 µm spot sizes were used. The whole field containing 27 × 27 spots was imaged using KEYENCE Fluorescence Microscope BZ-900 at 2× magnification, and then using the “merge” function of microscope software BZII-Analyzer at 0 h, 24 h and 48 h after seeding. 175 spots from each field were analysed. Cells in the droplets were counted manually using ImageJ. The images from different time points were aligned in order to be able to follow the content of each spot at all time points. Cells were considered viable if they were GFP positive and exhibited spread cell morphology. Cells were considered dead if they were GFP negative and exhibited a round morphology. The experiment was repeated 3 times independently, with 9 arrays analysed. 

### 2.5 Statistical Analysis

To study the distribution of cells inside the droplets on DMA, the number of cells in all droplets on the array was counted. DMA with 1 mm, 500 µm and 350 µm spots contained 196, 729 and 1521 droplets, respectively. The droplets were grouped depending on the amount of cells inside each droplet: 1 cell, 2 cells, 3 cells, 4 cells and 5 cells.

To study the proliferation and viability rate of cells on the DMA, 175 spots were analysed at 0, 24 and 48 h (the data from 48 h time point is presented in Supplementary Materials). Each analysis was performed based on combined data from 3 independent experiments. Two-tailed heteroscedastic *t*-test was used to determine if different sets of data significantly differ from each other. The analysis was performed in Microsoft Excel program. 

## 3. Results

### 3.1. Formation of the Single-Cell Droplet Microarray (SC-DMA)

**Figure 1:**
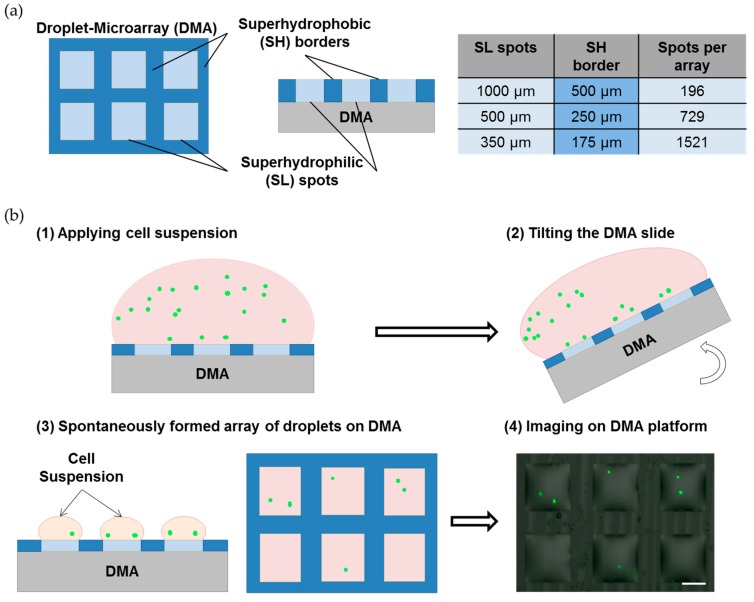
Single Cell Droplet Microarray (SC-DMA) platform. (**a**) Schematic illustration of a DMA slide and table showing the sizes of superhydrophilic (SL) spots, superhydrophobic (SH) borders and the corresponding number of droplets in a 2.5 cm × 2.5 cm DMA array; (**b**) The workflow of SC-DMA formation.

**Figure 2:**
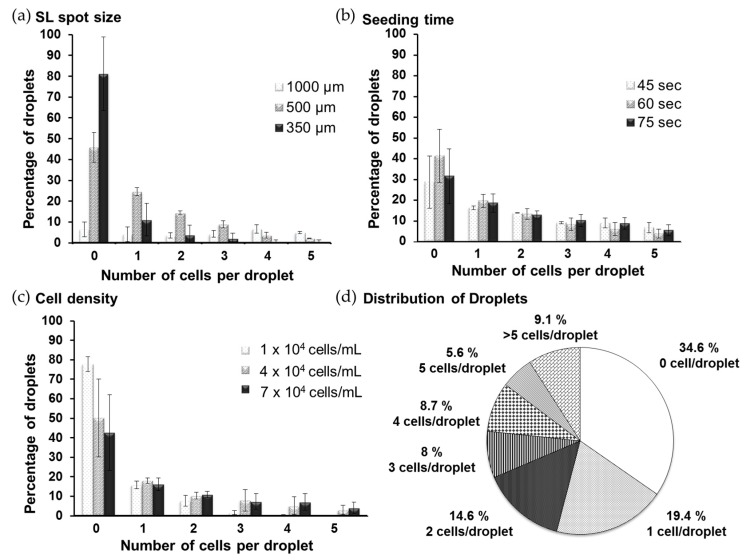
Distribution of cell numbers on SC-DMA. (**a**) Distribution of droplets with certain number of cells in relation to the size of SL spots; (**b**) seeding time; (**c**) and seeding density; (**d**) Distribution of droplets with different cell numbers, using standard condition (500 µm spot size, cell seeding density of 4 × 10^4^ cells/mL and 60 s seeding time). For all of the above experiments, *n* = 3.

In the current study, we used DMA containing 196, 729, 1521 square SL spots measuring 1 mm, 500 µm and 350 µm, respectively (Figure 1a). In order to seed cells on the DMA, we applied 1.4 mL of cell suspension onto the array for 40, 60 or 75 s, which we refer to as seeding time. Afterwards, we slightly tilted the slide, which resulted in the spontaneous formation of droplets containing cells (Figure 1b). To create the Single-Cell Droplet Microarray (SC-DMA), we followed the limiting dilution method. In this case, the distribution of single cells across the DMA follows a Poisson distribution, which depends on several parameters [37,38]. It is important to control these parameters in order to obtain the maximum possible number of droplets containing single cells. In order to achieve that, we tested three parameters to control the distribution of single cells across the DMA, which included different spot sizes (1 mm, 500 µm, and 350 µm), cell seeding density (1 × 10^4^ cells/mL, 4 × 10^4^ cells/mL, and 7 × 10^4^ cells/mL) and seeding times (45, 60, 75 s) (Figure 2). By testing and combining all of these parameters, we found optimal conditions that gave the highest number of droplets containing single cells: (1) 500 µm spot size; (2) 4 × 10^4^ cells/mL cell seeding density; and (3) 60 s seeding time. As shown in Figure 2d, by using these conditions, we were able to obtain SC-DMAs with at least 20.3% of the droplets containing single cells. We refer to these parameters as “standard condition” and use them for all further experiments on the SC-DMA. 

### 3.2. Viability and Growth Rate of Single Cells on the DMA Platform

**Figure 3:**
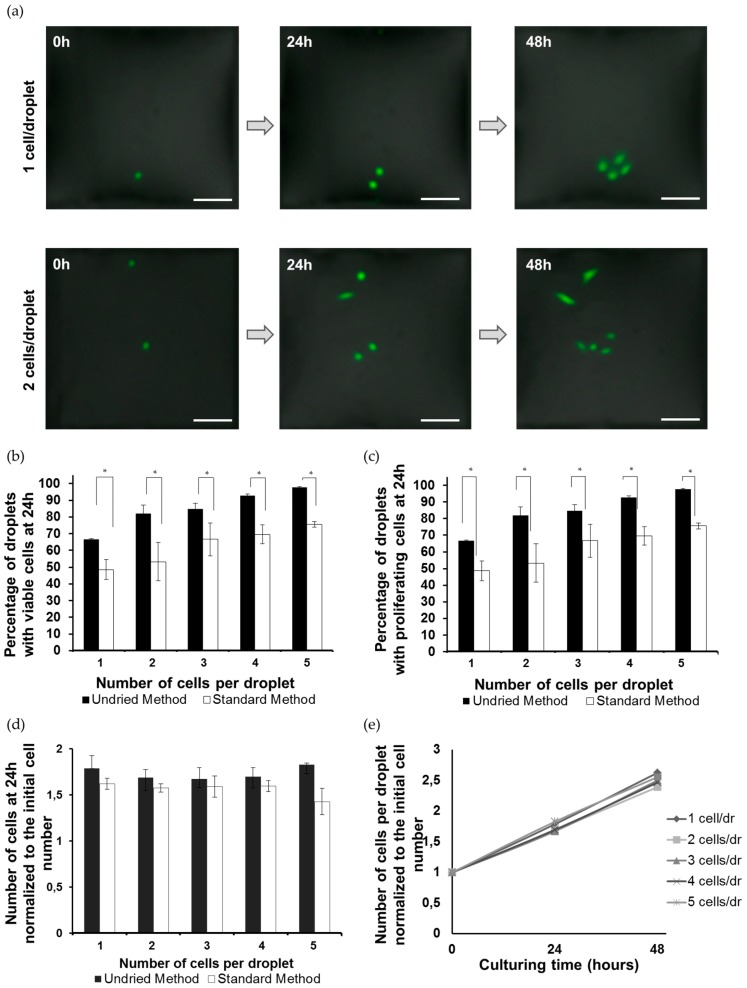
Cell viability and proliferation on the DMA platform. (**a**) Microscope images of two representative droplets containing HeLa-GFP with initial cell number of 1 (upper row) and 2 (lower row) cells per droplet. The droplets were imaged at 0, 24, and 48 h after seeding. Scale bar is 100 µm; (**b**) Percentage of droplets containing viable cells after 24 h of culturing using *undried* and *standard* method for preconditioning of the DMA; (**c**) Percentage of droplets containing proliferating cells after 24 h of culturing using *undried* and *standard* method for preconditioning of the DMA; (**d**) Comparison of proliferation rates of cells cultured in droplets with different initial cell number. For all of the above experiments, *n* = 3. * *p* < 0.05.

It is known that cells show lower viability and growth rate when cultured as single cells [39]. We estimated the viability and growth rate of cells on the DMA with spots measuring 500 µm, using the standard conditions to create SC-DMA. The whole array was imaged using 2× objective at different time points (0, 24, 48 h) after seeding (Figure 3 and Figure S1). The images were analysed by counting the number of cells per droplet, the content of the same droplet was monitored and counted over indicated time points (Figure 3a,b and Figure S1). We observed that 78.7% of cells cultured as a single cell were viable after 24 h of culturing (Figure 3b, “*standard* method”). There was no obvious difference in the percentage of droplets with viable cells after 24 h and 48 h of culturing when cells were cultured as a single cell or starting from two, three, four or five cells per droplet (Figure 3b and Figure S1a). This indicates a relatively high survival rate of single cells on the DMA, which is comparable with cultures starting with more than one cell. As a next step, we analysed the proliferation of cells in droplets containing one or more cells at the beginning of the culture. We showed that 48.7% of droplets containing single cells had proliferating cells within the first 24 h of culture, and 53.3%, 66.7%, 69.6%, 75.6% of droplets had proliferating cells in the case of cultures starting from two, three, four and five cells, respectively (Figure 3c). The increasing trend in the percentage of droplets containing proliferating cells with increasing initial cell numbers might be due to the biological advantages of cells when they are able to establish cell–cell communications. However, it can also be due to a higher probability of cell proliferation occurring in droplets with higher initial cell numbers. We compared the growth rate of cells in droplets containing one or more cells at the beginning of the culture by normalizing the number of cells at 24 or 48 h to the initial cell number (Figure 3d,e). We did not observe any significant difference in proliferation rates of cells between droplets initially containing different number of cells (Figure 3d,e and Figure S1b). This indicates that on the DMA platform single cells tend to proliferate at the same rate as cells in populations starting with more than one cell. 

Although culturing single cells on the DMA using the standard preparation method (see Section 2.1) provided a proper environment for cell survival and proliferation, we checked if the viability and proliferation of single cells on SC-DMA could be improved, first, by using different seeding medium and, second, by further optimizing the *standard* method for DMA preconditioning.

It is known that in cell populations cells release mediators, communicating with each other and promoting cell survival and proliferation [36,40,41]. Therefore, first, we checked if the use of medium enriched in such cell mediators can increase cell viability and proliferation rate. In order to enrich the medium with proteins and growth factors released by cells, we used the freshly collected (*20 h-*medium) or frozen (*20 h*-*frozen* medium) medium from HeLa-GFP cells cultured in Petri dish for 20 h as a culturing medium for SC-DMA. Our results showed that the use of both types of preconditioned media did not lead to an increase in the survival or proliferation of single cells (Figure S2). In contrast, it resulted in a decrease in the percentage of droplets containing (i) viable cells by 28% and 12% and (ii) proliferating cells by 28% and 27.3%, after using *20 h*- medium and *20 h*-frozen medium, respectively. A similar negative effect of the preconditioned medium on cell viability and growth was observed in droplets with more than one cell (Figure S2).

Additionally, we tested a different method of preconditioning the DMA slides prior to the cell seeding. We slightly changed the standard procedure that we used for conventional cell culturing on DMA. For the *standard* method, the DMA slides were preconditioned by incubation of the slides in medium containing 1% FCS for 25 min followed by 60 min air drying of the slides, coating of SL spots with gelatin and air drying the slides for 45 min before cell seeding. In the new method, which we refer to as *undried* method, we removed the drying step between the medium pre-incubation step and coating with gelatin and reduced the drying time after gelatin coating and before cell seeding from 45 to 20 min. As shown in Figure 3b,c, *undried* method resulted in an increase in the percentage of droplets containing live cells by 18%. This resulted in the survival of nearly all (96.7% for 24 h and 88% for 48 h) single cells (Figure 3b and Figure S1a). The percentage of droplets containing proliferating cells also increased from 48.7% and 49.3% to 66.7% and 80% at 24 and 48 h of culturing, respectively (Figure 3c and Figure S1b). We also observed a corresponding increase in cell viability and growth rate in droplets initially containing more than one cell (Figure 3b,c and Figure S1a,b). The doubling time of single cells within 24 h of culture across the whole single cell population considering the droplets containing non-proliferating cells was estimated to be 28.3 h, which is comparable with 23–24 h doubling time of HeLa cells in conventional culture setups. Thus, the optimized protocol increases the viability of single cells on the DMA platform to nearly 100% after 24 h of culturing. Cells proliferate at a rate comparable to that of cells cultured in bulk and conventional culturing conditions.

## 4. Discussion

In the current study, the methodology for single cell-analysis on the DMA has been established for the first time. The two main technical goals of this study were, first, to establish the optimal distribution of single cells across the DMA by optimizing parameters such as array size, cell seeding density and cell seeding time; and, second, to improve the survival and proliferation rate of single cells on the DMA. In addition, we investigated the correlation between the viability and growth of cells relation to the initial cell number in individual droplets on the DMA. 

By using the DMA platform and the seeding technique described in Figure 1b, we achieved stochastic Poisson distribution of cells across the DMA. We tried to control the distribution by varying the array size, cell seeding density and seeding time. Obtained results showed that the smaller (350 μm) and bigger (1 mm) array sizes did not lead to a favourable distribution of single cells on the DMA platform, whereas the DMA with 500 µm spot sizes enabled the best distribution that we could achieve using the developed methodology. At the same time, these conditions provided sufficient space and volume for single cells to live, proliferate and migrate within a droplet. Using the method of limited dilution, we optimized the final distribution of cells across the DMA by varying seeding time and cell density. We achieved a distribution of 20.3% of the droplets containing single cells, 43.5% with more than 1 cell and 36.2% empty droplets (Figure 2). The percentage of droplets occupied by single cells on the DMA platform is comparable or lower than those in some existing platforms used for single-cell analysis such as microarray chambers with ≈21% occupation of single cells per chamber and 384-well plates with ≈35% single cells per plate [42,43]. A high percentage of droplets containing more than one cell might be beneficial for some analyses, for example, to investigate differences in culture between one, two, three, four or five cells per droplet. However, the DMA platform is flexible and more parameters can be varied in order to achieve a better distribution of single cells by using the limited dilution method. Moreover, the DMA platform could be potentially used for dispensing the cells directly into SL spots, or in combination with microfluidic channel systems for the precise deposition of single cells into individual droplets. For the purpose of the current study, which demonstrates the potential of the DMA platform for single cell analysis and its advantages compared to existing technologies, the achieved single cell occupancy was sufficient.

The behaviour of cells in confined nanoliter reservoirs has not been widely explored. However, few studies suggested that cells have lower survival rates when cultured as single cells compared to being cultured in the form of cell populations [18]. Cell–cell interactions are essential for cells to provide necessary proteins and factors needed to support cell growth [44]. By using standard conditions, we obtained 78.7% of single cells surviving and 48.7% proliferating after 24 h of culture. In order to achieve higher survival and proliferation rates of single cells in individual droplets on the DMA platform, we changed the seeding medium and optimized the protocol for the preconditioning of the DMA slide.

The usage of different seeding media was tested given the assumption that the pre-incubated media might contain necessary proteins and factors and, therefore, might induce and enhance the proliferation and viability of single cells. However, our experiments showed that the *20 h*-medium and *20 h-frozen* medium led to a decrease in the proliferation and survival rate of single cells on DMA. This might be due to the depletion of nutrients and the accumulation of cytotoxic metabolites after 20 h of culture, resulting in impeded cell viability and proliferation. Further experiments have to be conducted to test if the medium collected after shorter culturing periods can stimulate the proliferation of single cells. 

We optimized the protocol for the preconditioning of the DMA slides. The drying process was shortened in the *undried* seeding method based on the assumption that it would result in a reduction of the evaporation rate of droplets on the DMA due to a higher viscosity coming from an undried gelatin layer. By using the *undried* method, we achieved an increase in the percentage of droplets containing viable single cells up to 96.7% and containing proliferating cells up to 66.7%. The doubling time of single cells for the first 24 h of culturing was on average 28.3 h, which is close to the 23–24 h doubling time of HeLa cells in conventional culture setups. In addition, we demonstrated that the viability and growth of cultures starting from a single cell did not differ from those of cultures started with 2–5 cells. Thus, we can conclude that single cells cultured in independent droplets on the DMA platform do not seem to behave differently from cells in bulk cell populations even comparing to conventional cultures. Considering that the survival rate of single cells on other platforms was shown to be lower (90% in microfluidic-based single-cell arrays [45]; 80% in droplet-based microfluidics [46]; ~60% in microtiter plates [47]), the DMA platform carries a clear advantage for culturing single cells. One of the possible reasons for this could be the small culturing volume where the growth factors and signalling molecules released by the cell are not diluted in a bigger volume, thus promoting a more favourable survival and growth of the cell itself. 

In addition, in terms of culturing conditions of single cells, the DMA platform offers general advantages compared to existing platforms which can be beneficial for performing high-throughput screening of single cells. DMA enables performing screening in droplets of 3–80 nL volumes, resulting in a several magnitude reduction of the reagent consumption. In addition, small volumes could be beneficial in obtaining the material from the cell content, such as DNA or RNA. The main limitation of working with single cells lies in the insufficient amount of material for analysis that can be achieved from a single cell. This is mainly due to the loss of material during the isolation procedures, which are performed in very large volumes and require multiple washing and transfer steps. DMA can enable obtaining the cell material directly in miniaturized droplets reducing the risk of losing the material during the procedure. Finally, any reactions based on affinity binding, such as hybridization or antibody-based assays, are likely to be more sensitive in small volumes due to constricted spatial distribution of the molecules compared to that in large volumes. Another advantage of the DMA platform is that it can be operated in a pipetting-free, high-throughput manner and allows for easy spreading, treatment, staining, fixation and microscopic analysis of the cells [34,35]. For example, the whole DMA slide containing thousands of droplets can be scanned within several minutes to analyse the distribution of single cells in the droplets. Cell morphology and migration can be easily observed by simple microscopy. The DMA platform is compatible with conventional automated and manual microscopes and does not require any special equipment to operate. In addition, the DMA platform is an open system and cells could be retrieved from the droplets in case it is required by the protocol. On the other hand, it provides a complete isolated and confined environment for cells to grow where the influence of environmental factors can be precisely controlled. Thus, the DMA platform can be used for a broad spectrum of applications related to SCA including fundamental research on homogeneity of cell populations and single cell behaviour, clone selection and high-throughput screening of single cells in combination with various read-outs, which could be based on microscopy or mass spectrometry analysis. We believe that the DMA platform is a novel and potent tool for SCA that can be easily used by the research community.

## 5. Conclusions

In the current study, we demonstrated the usefulness and advantages of the Droplet Microarray platform for single cell analysis. We optimized the distribution of single cells across the Droplet Microarray using the limited dilution method. We optimized the culturing conditions of single cells obtaining nearly 100% viability and a proliferation rate close to that of HeLa cells cultured by conventional methods. We did not observe any difference in proliferation and viability rates in culture of single cells versus culture with more than one cell, starting from two to five cells. Thus, we can conclude that single cells cultured in individual droplets do not seem to behave differently from cells in bulk populations even comparing to conventional cultures. The simplicity of operation of the Droplet Microarray platform, including pipetting-free spreading, treatment, staining and fixation of cells, compatibility with conventional microscopy analysis and versatility of the platform for various cell-based assays, make this technology a potent tool for single-cell analysis. 

## Supplementary Materials

The following are available online at www.mdpi.com/2076-3905/5/4/28/s1, Figure S1: Proliferation and Viability of cells on the DMA platform; Figure S2: Cell viability in droplets across the DMA in regards to different seeding media.
