# Supplementary material for: Droplet Microarray Based on Superhydrophobic-Superhydrophilic Patterns for Single Cell Analysis
Source: Microarrays (Basel). 2016 Dec 9;5(4):28. doi: 10.3390/microarrays5040028 (PMC5197947; doi:10.3390/microarrays5040028)
Supplement: Supplementary file 1 [file microarrays-05-00028-s001.pdf]

Supplementary Material: Droplet Microarray Based on Superhydrophobic-Superhydrophilic Patterns for Single Cell Analysis

Gabriella E. Jogia, Tina Tronser, Anna A. Popova and Pavel A. Levkin

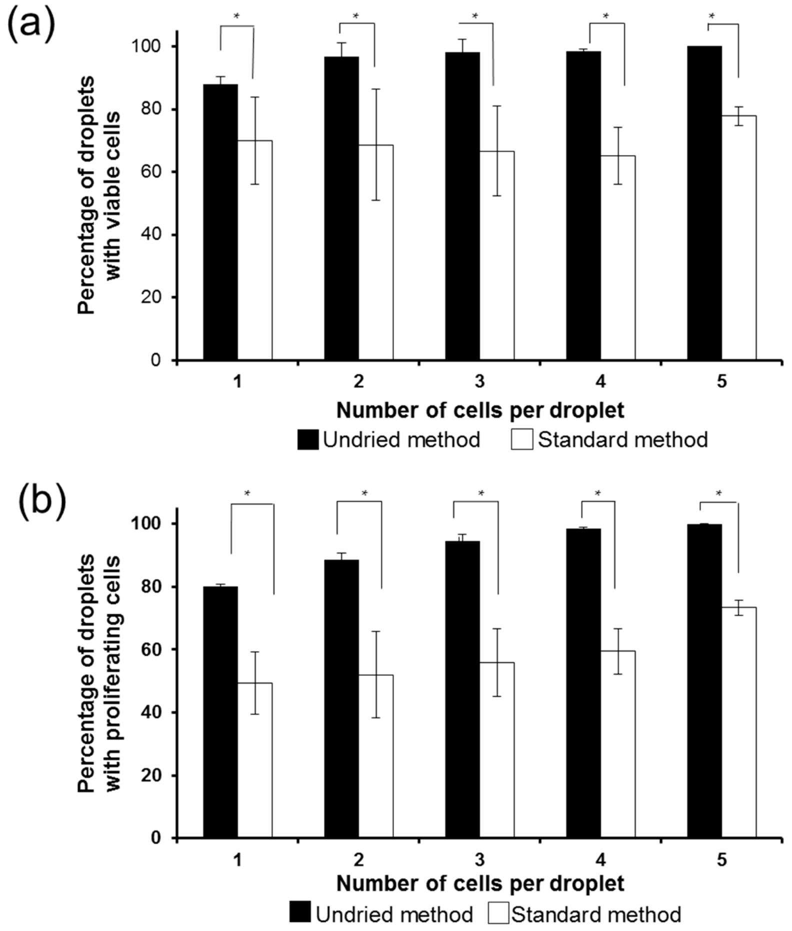

Figure S1. Viability and proliferation of cells on the DMA platform. (**a**) Percentage of droplets containing viable cells at 48 h of culturing using the undried and standard method for the preconditioning of the DMA. (**b**) Percentage of droplets containing proliferating cells at 48 hours of culturing using the undried and standard methods for the preconditioning of the DMA. For all of the above experiments, $n = 3$.

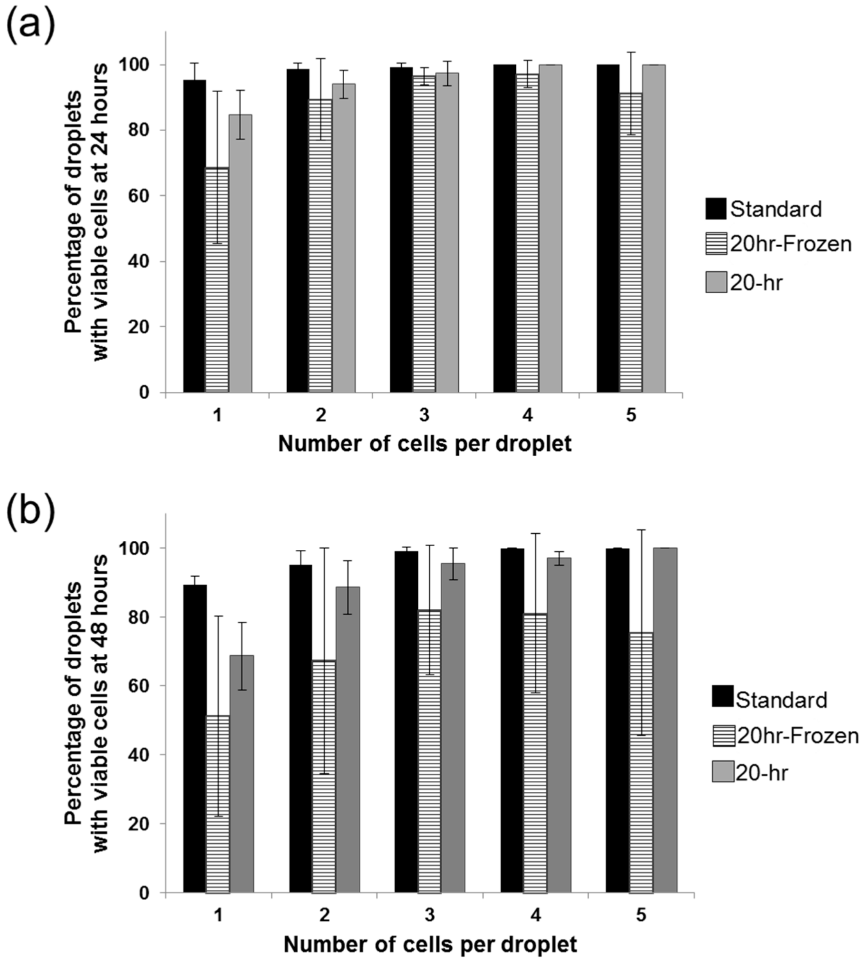

Figure S2. Cell Viability in droplets across the DMA in relation to different seeding media. Graph shows changes in viability of cells in droplets with certain number of cells when cultured using standard medium (black), 20 h-frozen medium (stripes) and 20 h-medium (grey) at (**a**) 24 h and (**b**) 48 h. For all of the above experiments, $n = 3$.
